# Bridging the treatment gap in India: Online training of psychologists in basic mental health services

**DOI:** 10.1192/j.eurpsy.2021.2199

**Published:** 2021-08-13

**Authors:** A. Kapanee, P. Sudhir, L. Suman

**Affiliations:** Clinical Psychology, National Institute of Mental Health and Neuro Sciences (NIMHANS), Bengaluru, India

**Keywords:** community mental health, Psychologists, online training, mental health treatment gap

## Abstract

**Introduction:**

The National Mental Health Survey of India 2015-16 (Gururaj et al., 2016) indicated a large treatment gap of 70-92% for mental disorders and a paucity of mental health specialists in the country. In order to address this treatment gap and develop human resources, the National Institute of Mental Health and Neuro Sciences (NIMHANS), Bengaluru, India, with impetus from the Ministry of Health and Family Welfare, Govt. of India, launched the online course of Diploma in Community Mental Health for Psychologists.

**Objectives:**

The course was designed with the objective of training individuals with a Master’s Degree in Psychology, in providing first-level psychological care in the community.

**Methods:**

The course is a 3-month online programme comprising of approximately 25 hours of self-paced e-learning and 11 hours of live real-time interactive discussion via video conference. The course comprises of 6 modules, with an assessment at the completion of each module. Pre- and Post-Assessment is conducted to evaluate competencies achieved.

**Results:**

On successful completion of the course, trainees are expected to have achieved competencies to: Screen for and identify mental health problems in adults and children, and understand factors influencing them; Understand management options; Conduct interview-based functional developmental assessment for intellectual deficits; Conduct first-level brief psychosocial interventions; Make appropriate referrals to Mental Health Professionals and other health professionals.

**Conclusions:**

This digitally-driven online course is a viable option for development of human resources on a large scale, in a resource-scarce (i.e. of mental health specialists) country such as India.

**Disclosure:**

No significant relationships.

